# Post-Translational Regulation of Oct4 Transcriptional Activity

**DOI:** 10.1371/journal.pone.0004467

**Published:** 2009-02-16

**Authors:** Jonathan P. Saxe, Alexey Tomilin, Hans R. Schöler, Kathrin Plath, Jing Huang

**Affiliations:** 1 Department of Molecular and Medical Pharmacology, David Geffen School of Medicine, University of California Los Angeles, Los Angeles, California, United States of America; 2 Institute of Cytology, Russian Academy of Science, St. Petersburg, Russia; 3 Department of Cell and Developmental Biology, Max Planck Institute for Molecular Medicine, Münster, Germany; 4 Department of Biological Chemistry and Broad Center for Regenerative Medicine, University of California Los Angeles, Los Angeles, California, United States of America; Baylor College of Medicine, United States of America

## Abstract

Oct4 is a key component of the molecular circuitry which regulates embryonic stem cell proliferation and differentiation. It is essential for maintenance of undifferentiated, pluripotent cell populations, and accomplishes these tasks by binding DNA in multiple heterodimer and homodimer configurations. Very little is known about how formation of these complexes is regulated, or the mechanisms through which Oct4 proteins respond to complex extracellular stimuli which regulate pluripotency. Here, we provide evidence for a phosphorylation-based mechanism which regulates specific Oct4 homodimer conformations. Point mutations of a putative phosphorylation site can specifically abrogate transcriptional activity of a specific homodimer assembly, with little effect on other configurations. Moreover, we performed bioinformatic predictions to identify a subset of Oct4 target genes which may be regulated by this specific assembly, and show that altering Oct4 protein levels affects transcription of Oct4 target genes which are regulated by this assembly but not others. Finally, we identified several signaling pathways which may mediate this phosphorylation and act in combination to regulate Oct4 transcriptional activity and protein stability. These results provide a mechanism for rapid and reversible alteration of Oct4 transactivation potential in response to extracellular signals.

## Introduction

The use of embryonic stem cells as therapeutics requires firm understanding of the mechanisms that control their proliferation and differentiation. To date, much progress has been made towards identifying extrinsic and intrinsic regulators of these processes. Studies have identified transcription factors such as Stat3, Nanog, and Oct4 as being necessary for embryonic stem (ES) cell self-renewal and maintenance of pluripotency. Likewise, it has been shown that signaling pathways and transactivation potentials triggered by extracellular stimuli such as BMPs, LIF, and other factors play major regulatory roles (for review see [1], [2]). For instance, the role of LIF-gp130-Stat3 axis in promoting ES cell proliferation is particularly well-defined [1], [3], and BMP-induced differentiation signals are inhibited by a Nanog-Smad1 protein complex [4]; crosstalk between these pathways has also been reported [5]. Several recent studies have shed light on transcriptional networks controlled by factors such as Oct4 (for instance, [6], [7]), and begun to address the issue of how extracellular cues are integrated with transcriptional circuits that maintain the pluripotent state [8]. Despite these findings, however, it is generally not clear how extrinsic cues are integrated within the cell to control the behavior of cell-intrinsic regulators of ES cell pluripotency such as Oct4 [1].

Oct4 is a transcriptional regulator that can either activate or repress target gene expression, depending on the cellular context [9], [10]. Oct4 messenger RNA is present in fertilized oocytes and early embryos, and expression is maintained until mid-gastrulation at which point it disappears, with the exception of primordial germ cells and their progeny [11], [12]. Oct4 expression is necessary for the establishment of the inner cell mass of the blastocyst [13], and proper levels of Oct4 expression are critical for maintenance of pluripotency [14]. Using engineered ES cells, it has been shown that increases or decreases of more than 50% of wild-type Oct4 mRNA levels is sufficient to induce differentiation towards embryonic or trophectodermal lineages, respectively [14]. This pattern is complemented by the phenotypes observed following decreased Nanog expression; ES cells differentiate towards endodermal fates upon Nanog loss-of-function [15], suggesting that combinatorial functions of multiple proteins contribute to the maintenance of pluripotency of ES cells partially through inhibition of differentiation [16].

Oct4 has two distinct DNA binding domains which independently bind half-sites of the canonical octamer motif. This flexibility allows Oct4 to form heterodimers with other transcription factors and to form homodimers in several conformations, depending on the configuration of the octamer half-sites within the DNA motif [17]. It has been shown that two such homodimers assemble using distinct, mutually exclusive interaction faces [18]. Hence, a potential phosphorylation event might be able to prevent formation of one of these conformations while leaving the other homodimer (as well as heterodimer formation potential) intact. As all of these Oct4 homodimer and heterodimer conformations bind distinct DNA motifs, a signaling-based mechanism could potentially control the transcription of distinct subsets of Oct4 target genes.

Through this mechanism, it would be possible for a cell to couple extracellular cues to maintenance of pluripotency through direct regulation of transcription factor activity, and to fine-tune gene expression as the extracellular environment dictates. Here, we provide evidence for such a phosphorylation-based mechanism. Mutation of a potential protein kinase A (PKA) phosphorylation site has dramatic consequences on Oct4 transactivation potential. Surprisingly, small molecule activators of PKA signaling increase expression of Oct4 protein, which in turn enhances expression of a specific subset of Oct4 target genes. These effects are mediated at least in part via p38 MAP kinase, thereby providing multiple means for rapid control of Oct4 transactivation in response to complex extracellular stimuli throughout early development.

## Results

### Regulated degradation of Oct4 protein

**Figure 1:**
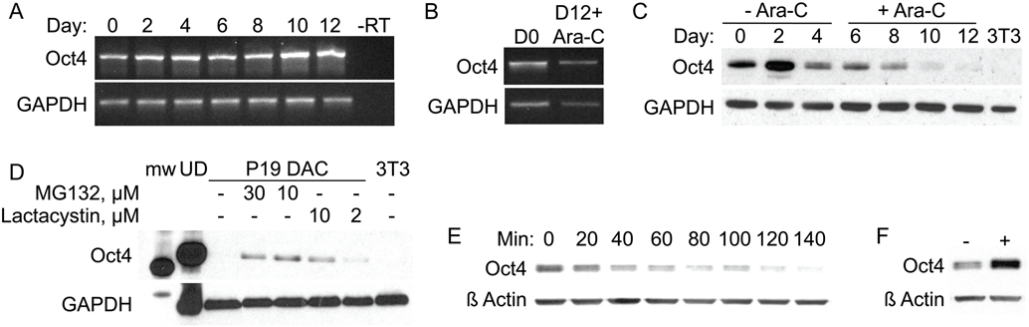
Oct4 protein is regulated via controlled degradation. (A) P19 cells were differentiated by aggregate formation in the presence of DMSO (as described in Methods) for the indicated number of days, followed by RT-PCR analysis of Oct4 expression. (B) RNA was collected from untreated P19 cells or cells differentiated as described above with the addition of 5 ug/mL cytosine arabinosidase (Ara-C), followed by RT-PCR analysis. (C) P19 cells differentiated as above, in the presence of Ara-C and analyzed for expression of Oct4 protein. (D) Differentiated Ara-C treated P19 cells were treated with indicated doses of proteasome inhibitors, followed by western blot analysis of Oct4 protein expression. (E) Undifferentiated P19 cells were treated for indicated times with 20 ug/mL cyclohexamide, followed by western blot analysis of Oct4 expression. (F) Undifferentiated P19 cells, untreated or treated with 10 uM MG-132 for 1 hour, followed by western blot analysis.

Undifferentiated embryonic stem (ES) cells express Oct4 mRNA and protein; this expression is rapidly downregulated during embryoid body formation. Likewise, P19 cells induced to differentiate via aggregation in the presence of retinoic acid also turn off Oct4 expression rapidly following induction [19]. Substitution of dimethyl sulfoxide (DMSO) for retinoic acid during P19 cell aggregation results in appearance of various mesodermal cell types [20]. It was expected that differentiation with DMSO would likewise cause a reduction in Oct4 mRNA levels. Differentiation was induced as described in *Materials and Methods*, and aggregates were plated and differentiated for an additional eight days. Identical to previous reports [21], cultures contained cells characteristic of DMSO-differentiated P19 cells (data not shown). Oct4 mRNA expression was analyzed by semi-quantitative RT-PCR. Surprisingly, however, Oct4 mRNA levels did not decrease during differentiation with DMSO (Figure 1A). To eliminate the possibility of any undifferentiated cells in the cultures accounting for this observation, DMSO-differentiated cells were treated with the anti-proliferative agent cytosine arabinosidase (Ara-C), starting two days after aggregate plating. Six days after the start of Ara-C treatment, cultures expressed Oct4 mRNA at levels similar to undifferentiated cells (Figure 1B) and exhibited morphology characteristic of endodermal cell types (data not shown).

Analysis of Oct4 protein expression in differentiated Ara-C cultures (DACs) showed a slight increase of Oct4 protein by day four of aggregate formation (Figure 1C), consistent with previous reports examining Oct4 mRNA dynamics [22] and the finding that primitive endoderm differentiation is accompanied by a transient increase in Oct4 protein levels [23]. Following aggregate plating and Ara-C addition, Oct4 protein expression progressively decreased, disappearing by day twelve. As these cultures still express Oct4 mRNA (Figure 1A), we asked whether they are synthesizing new Oct4 protein. We reasoned that if these cultures are synthesizing new Oct4 protein, it must be rapidly degraded, possibly in a proteasome-dependent manner [24]. Therefore, twelve day old, DMSO-differentiated, Ara-C treated P19 cultures were incubated with the proteasome inhibitors MG-132 and lactacystin for four hours; this was sufficient to rescue Oct4 protein expression (Figure 1D), suggesting that these cells do indeed synthesize new Oct4 protein.

We then asked whether undifferentiated P19 cells also exhibit this tight regulation of Oct4 protein levels. Cyclohexamide-induced block of new protein synthesis shows that Oct4 protein has a half-life of approximately 90 minutes in these cells (Figure 1E), and treatment with 10 µM MG-132 for an hour resulted in a significant increase in Oct4 protein levels (Figure 1F). Hence, turnover of Oct4 protein is a dynamic process, with new protein constantly replenishing older pools as they are degraded. Previous work has shown that precise levels of Oct4 transcript and protein are required for ES cell maintenance; raising or lowering levels within a narrow window is sufficient to induce differentiation or de-differentiation, respectively [14].

### Phosphorylation at serine 229 partially controls Oct4 transactivation activity

**Figure 2:**
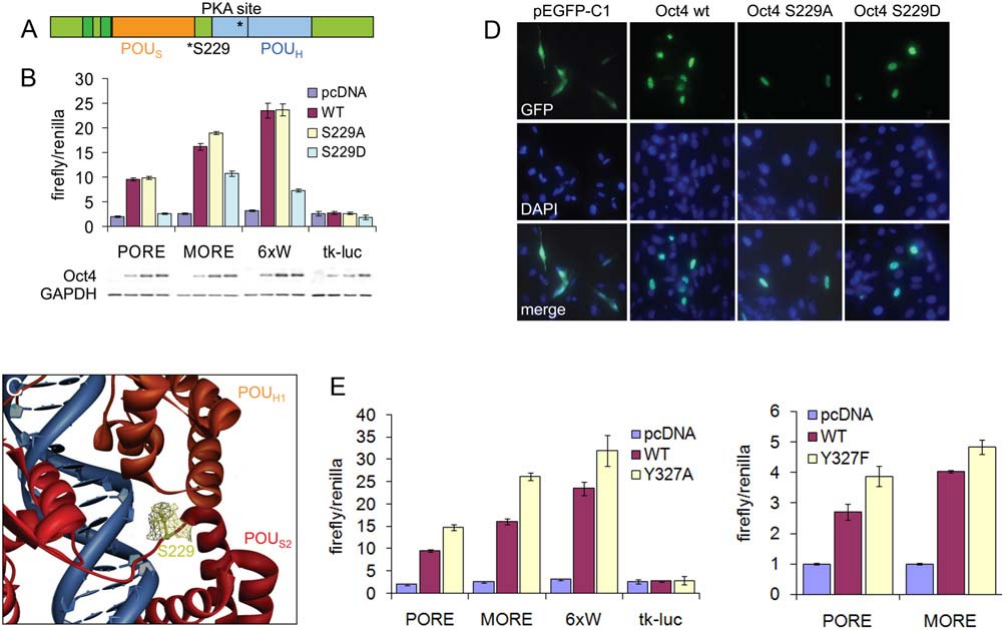
Control of Oct4 dimerization via putative phoshorylation sites. (A) Schematic representation of Oct4 protein, highlighting the predicted protein kinase A (PKA) site at S229. (B) Luciferase assays in 3T3 cells demonstrating activity of Oct4 point mutants on activation of different reporter constructs. Data is normalized to CMV-renilla luciferase activity. (C) S229 phosphorylation of Oct4 can cause steric and electrostatic clashes with the other Oct4 molecule in the PORE homodimer and with DNA binding. Crystal structure is PDB 1HF0. (D) Oct4 mutants fused to GFP do not mis-localize to the cytoplasm. Cells were transfected with indicated constructs, and counterstained with DAPI and imaged 48 hours later. (E) Luciferase assays as in (B) demonstrating the effects of mutation of Y327 to alanine or phenylalanine on indicated reporter constructs.

One implication of these findings is that Oct4 may be subject to post-translational modifications which alter its activity. To identify sites for such modifications, we searched for potential regulatory motifs using bioinformatic prediction algorithms (http://scansite.mit.edu, [25]) and identified a putative protein kinase A (PKA) phosphorylation site at S229 within the POU_S_ domain of Oct4 (Figure 2A). As this residue lies on the edge of the POU homeodomain, we asked if phosphorylation could influence the transactivation potential of Oct4.

Flag-tagged point mutants which mimic (S→D) or prevent (S→A) phosphorylation at this site were generated and co-transfected into NIH 3T3 cells with Oct4 luciferase reporter constructs. These constructs are activated by Oct4 monomers (6xW) or homodimers in one of two distinct configurations (PORE and MORE, due to different arrangements of the octamer sequences within these motifs, see Discussion for details [10], [26]). As shown in Figure 2B, the S229A mutant behaved similar to WT Oct4 (pcDNA3 vs WT, p<0.001 on PORE, MORE, and 6xW; WT vs S229A, p>0.05 on PORE and 6xW, p<0.05 on MORE). In contrast, the S229D mutant was unable to transactivate the PORE reporter, but retained ability to activate (albeit at lower levels) the other Oct4 reporters (pcDNA3 vs S229D, p>0.05 on PORE, p<0.001 on MORE and 6xW; WT vs. S229D, p<0.001 on MORE, PORE, and 6xW) . These differences were not due to changes in nuclear localization as tested by the distribution of GFP fusions of these mutants (Figure 2D, an important consideration as the PKA site lies on the edge of an identified nuclear localization sequence [27]). Previously determined crystal structures of a similar Oct-1 DNA binding domain have demonstrated that the analogous residue is in close proximity to the interface between the two molecules of the homodimer and DNA (and that this model is conserved in Oct4 homodimers, [18]), suggesting that phosphorylation at this residue may sterically hinder both DNA binding and homodimer assembly (Figure 2C).

Also identified through this analysis was a potential Abl kinase site at Y327 of Oct4. Mutation of this tyrosine to alanine or phenylalanine resulted in hyperactive transactivation on all reporter constructs tested (Figure 2E, S229A vs. Y327A, p<0.001 on PORE and MORE, p<0.05 on 6xW; S229A vs. Y327F, p<0.05 on PORE and MORE), suggesting that this site may act to fine-tune Oct4 transcription through an undefined mechanism.

### Identification of a cohort of Oct4 target genes regulated via a cAMP-responsive pathway

**Figure 3:**
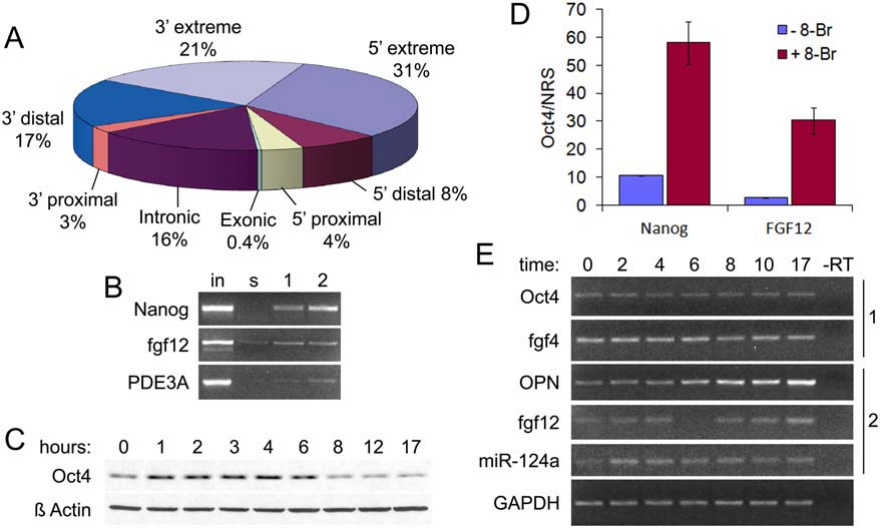
Specific regulation of a sub-set of Oct4 genes regulated by the PORE sequence. (A) BLAST analysis of the PORE sequence reveals hundreds of potential binding sites within the mouse genome. Sites are organized by distance from known genes: extreme, >100 kb; distal, between 10–100 kb; proximal, between 0–10 kb; intronic and exonic, within known genes. (B) Validation of fgf12 and PDE3A as bona fide Oct4 targets by chromatin immunoprecipitation with either serum (s, control) or monoclonal (1) or polyclonal (2) antibodies against Oct4. Nanog, positive control. (C) Undifferentiated P19 cells were treated with 8-Br-cAMP for the indicated times and analyzed by western blotting. (D) Treatment with 1 mM 8-Br-cAMP for four hours enhances Oct4 binding to genomic loci as determined by chromatin immunoprecipitation, followed by real-time PCR analysis at Nanog and fgf12 sites. Data is represented as the ratio of % precipitated DNA of input following precipitation with Oct4 antibody or rabbit serum control, ±SD. (E) Undifferentiated P19 cells were treated with 1 mM 8-Br-cAMP for indicated times. Expression of multiple Oct4 target genes controlled by Oct4/Sox2 heterodimers (1) or PORE-configuration homodimers (2) were assayed by RT-PCR.

The fact that phosphorylation of Oct4 at S229 abolishes its transactivation potential on the PORE sequence, but not other octamer motif configurations, raises the possibility that this may be a mechanism to regulate expression of those Oct4 target genes controlled by a PORE sequence. However, only one gene with such a sequence (osteopontin) is known to be an Oct4 target gene [10]. To determine if this model could truly be biologically relevant, we determined the extent of PORE sequence occurrence in the mouse genome by BLAST analysis. The PORE sequence is 15 nucleotides long; therefore, the chance of it randomly occurring is approximately 1/1,000,000,000 ( = 4^15^). Thus, this sequence could occur by chance about three times in the 2.7×10^9^-bp mouse genome. We found 652 exact, distinct occurrences in NCBI mouse genome build 37.1. 411 of these matches were located within 250 kb of 348 annotated genes. Of these 411 matches, 156 were 10–100 kb away from annotated genes, 41 were located between 10 kb and the gene boundary, and 129 were found within the genic sequence (Figure 3A, Table S1).

Upon filtering the list of 348 genes against lists of target genes derived from whole-genome analysis studies of Oct4 binding [6], [28], 30 PORE genes were found to be *bona fide* Oct4 targets (Table S2). Gene ontology (GO) analysis of genes within 250 kb of a PORE sequence revealed enrichment in processes such as transcription regulator activity (p<0.001), sex determination (p<0.005), insulin receptor signaling (p<0.001), development (p<0.0005), and protein phosphorylation (p<0.005). Binding of Oct4 to several predicted PORE targets was verified via chromatin immunoprecipitation (ChIP, Figure 3B). Thus Oct4 binding to the PORE sequence is not an isolated event, and regulation of Oct4 binding specifically to this motif could be a major mechanism of transcriptional control of stem cell self-renewal pathways.

**Figure 4:**
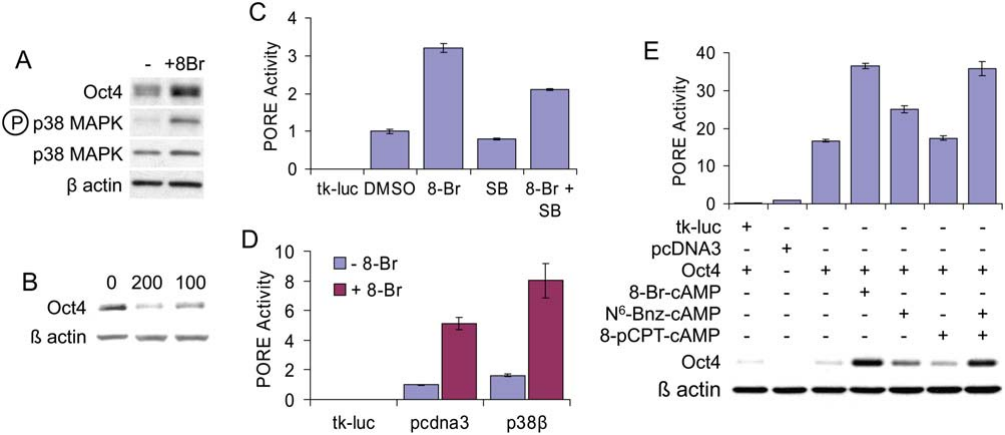
Regulation of Oct4-PORE activity by an 8-Br-cAMP-responsive pathway coupled to p38 MAP kinase. (A) P19 cells treated with 1 mM 8-Br-cAMP for 1 hour followed by analysis of Oct4 expression and p38 MAP kinase phosphorylation. (B) Treatment of P19 cells with indicated nM concentrations of the p38 MAP kinase inhibitor SB202190 for 30 minutes, followed by western blotting. (C) P19 cells were transfected with control vector (tk-luc) or PORE reporter, followed by overnight treatment with indicated combinations of 0.5 mM 8-Br-cAMP and 200 nM SB202190. (D) Luciferase assay in P19 cells following transfection with tk-luc or PORE reporters in combination with empty vector (pcDNA3) or p38β MAPK expression vector, and overnight treatment with or without 0.5 mM 8-Br-cAMP. (E) Luciferase assay and western blot analysis of 3T3 cells following transfection with PORE reporter and overnight treatment with indicated cAMP analogues.

We then tested whether PKA signaling can regulate transcription of PORE genes. Treatment of P19 cells with the PKA activator 8-Br-cAMP resulted in a large, rapid (<1 hour), and transient (<8 hours) increase in Oct4 protein levels (Figure 3C). This increase was accompanied by enhanced transactivation of Oct4 luciferase reporters (Figure 4C) and increased chromatin occupancy by Oct4 at native PORE (fgf12, p<0.01) and non-PORE (Nanog, which is regulated by an Oct4/Sox2 heterodimer, p<0.005) target genes (Figure 3D). The consequences of this occupancy were then determined by RT-PCR analysis of a set of Oct4 target genes following 8-Br-cAMP treatment. Provocatively, genes controlled by Oct4 heterodimers (Oct4 itself, fgf4 [6], [13]) showed either no increase or a slight decrease in transcript levels, whereas those controlled by PORE homodimers (fgf12, osteopontin, mIR-124a) generally displayed enhanced transcription (Figure 3E and Figure S1, albeit with distinct kinetics).

### p38 MAP kinase functions downstream of PKA to regulate Oct4 activity

Brief stimulation with 8-Br-cAMP was sufficient to enhance Oct4 protein levels; this enhancement was accompanied by activation of the p38 MAP kinase pathway (Figure 4A), as measured by western blotting. To test if enhanced p38 signaling was simply correlated with or actively controlled levels of Oct4 protein, P19 cells were treated with the p38 MAPK inhibitor SB202190. Inhibition of p38 activity resulted in a large and rapid decrease in Oct4 protein levels (Figure 4B). In PORE reporter assays in P19 cells, treatment with SB202190 alone caused a slight, though statistically non-significant, decrease in reporter transactivation; however, SB202190 was able to blunt 8-Br-cAMP-induced reporter activation by ∼33% (Figure 4C; p<0.001). Furthermore, overexpression of p38β MAP kinase enhanced 8-Br-cAMP-induced Oct4 transcriptional activity (Figure 4D; 8-Br-treated pcDNA3 vs. 8-Br-treated p38β, p<0.05 ).

The discovery of EPAC as an additional intracellular cAMP receptor [29], the finding that cAMP-induced PKA (but not EPAC) signaling is coupled to the p38 MAPK pathway in some cell types [30], and the observation that SB202190 partially, but not completely, inhibited 8-Br-cAMP-stimulated Oct4 activity (Figure 4D) led us to ask whether all of the 8-Br-cAMP response was mediated through PKA/p38 MAPK. To address this issue, highly specific cAMP analogues were used. N^6^-Bnz-cAMP is highly selective for PKA and does not activate EPAC. Conversely, 8-pCPT-O-Me-cAMP is selective for EPAC but does not stimulate PKA [31]. In NIH 3T3 cells transfected with PORE reporter and Oct4 expression constructs, overnight treatment with N^6^-Bnz-cAMP elicited a response ∼50% of that obtained by treatment with 8-Br-cAMP, at both the transcriptional (p<0.001 vs. untreated and vs. 8-Br-cAMP) and protein levels (Figure 4E). Treatment with 8-pCPT-O-Me-cAMP, in contrast, had no effect on Oct4 reporter transactivation (p>0.05, untreated vs. 8-pCPT). Surprisingly, co-treatment with N^6^-Bnz-cAMP and 8-pCPT-O-Me-cAMP elicited an effect equal to that of 8-Br-cAMP (Figure 4E; 8-Br-cAMP vs. N^6^+8-pCPT, 37.28±1.26 vs. 32.60±3.13, p>0.05; N^6^ vs. N^6^+8-pCPT, p<0.001), suggesting a synergistic effect between PKA and EPAC activation on Oct4 transcriptional activity and protein stability.

## Discussion

In this paper, we describe a mechanism for regulation of a subset of Oct4 target genes. Although transcriptional networks controlled by Oct4 have been delineated [6], [28], [32] and several protein interactors of Oct4 have been identified [24], [33], the mechanisms through which Oct4 protein itself is regulated have largely remained unexplored.

### Mechanisms for maintenance of steady-state levels of Oct4 protein

Here, we exploit an unusual, previously noted property of DMSO-differentiated P19 cells, namely the de-regulation of Oct4 transcription upon differentiation. It is well established that Oct4 mRNA and protein disappear relatively quickly following differentiation of embryonic stem (ES) cells, by multiple protocols. Similar results have been shown upon retinoic acid-induced differentiation of P19 cells ([34], although re-appearance of low levels of Oct4 mRNA [22] and protein [35] have been noted).

In contrast, previous studies have shown that Oct4 mRNA levels can remain relatively stable during DMSO-induced differentiation of P19 cells, although this has been ascribed to undifferentiated, highly proliferative cells present in the cultures following differentiation [22], [34]. Here, we also observed little change in Oct4 mRNA levels following DMSO-induced differentiation, and we show that the transcript is present in differentiated, non-proliferative cells (Figure 1B). Silencing of the Oct4 promoter in P19 cells is a complex process, mediated by a combination of events including orphan receptor binding, DNA methylation, histone deacetylation, and heterochromatin formation [36], [37]. It is possible that the Oct4 expression patterns that we observe is due to failure of one or more of these processes in the complex, abnormal milieu of this embryonal carcinoma cell line during differentiation.

Regardless, we were able to utilize this property to study regulated turnover of Oct4 protein and further showed that our results applied to undifferentiated, steady-state levels of Oct4. Ubiquitination of Oct4 by a specific E3 ubiquitin ligase, with resultant proteasomal degradation, has previously been described [24]. Here, we confirm proteasomal turnover of intracellular Oct4 pools and also show that new Oct4 protein is continually produced. As ES cells are exquisitely sensitive to changes in Oct4 protein levels [14], this directly suggests that there must be an active mechanism for marking existing Oct4 protein for degradation and raises the possibility of post-translational modifications in regulation of Oct4 levels and, by extension, maintenance of ES cell pluripotency.

### Post-translational regulation of Oct4 may activate a distinct set of target genes

As a first step towards identifying such post-translational modifications, we performed bioinformatic analysis of the Oct4 protein sequence and identified several potential phosphorylation sites. Previous studies have shown that the closely related Oct1 is dynamically phosphorylated [38], and site-directed mutagenesis of potential phosphorylation sites in Oct4 indicated a potential role for phosphorylation in regulating homodimer complex formation [18]. Oct4 contains two distinct DNA binding domains (the POU_H_ and POU_S_ domains, each of which can bind to half of the DNA octamer motif independently of the other) which are separated by a flexible linker [39]; this in turn allows for substantial leeway in arrangement of these domains in relation to each other [17]. Oct4 can bind to the Octamer motif (ATGCAAAT) as a monomer (in the Results section, this is referred to as the 6xW reporter) or as a heterodimer with several different proteins. In contrast, the PORE sequence (*P*alindromic *O*ct factor *R*ecognition *E*lement, ATTTGAAATGCAAAT) was originally identified in the first intron of osteopontin (OPN) and cooperatively binds two Oct4 molecules; binding sites for additional transcription factors such as Sox2 and Engrailed were identified in close proximity [10]. Later analysis of Octamer protein binding specificities revealed an additional DNA sequence, the MORE (*M*ore P*ORE*, ATGCATATGCAT), which likewise binds two Oct4 molecules. As described in Tomilin et. al., the key difference between the PORE and MORE sequences is that the two DNA binding domains of Oct4 (the POU_H_ and POU_S_ domains) which bind the octamer half-site originate from the same protein molecule when bound to the PORE; in contrast, one protein molecule contributes the POU_H_ domain while the other homodimer molecule contributes the POU_S_ domain when binding to the octamer half-site in the MORE configuration [26].

Thus, depending on arrangements of the octamer motif, and presence of DNA motifs for additional transcription factors, Oct4 can form heterodimers with multiple partners or homodimers in one of several unique conformations [17]. As detailed above, these different conformations have corresponding consensus sequences [10], [26], and the Oct4 monomers interact with each other via distinct protein faces depending on this sequence [18]. Hence, phosphorylation (or other modifications) of sites on one interaction face could potentially prevent DNA binding by one homodimer configuration, while leaving binding of other configurations intact.

We found that mutation of serine 229 to aspartic acid, which mimics phosphorylation at this site, does indeed prevent Oct4 transactivation potential of one homodimer conformation but not of other Oct4 complexes. Based on the crystallographic structure of the Oct1 DNA-binding domain (to which the Oct4 domain is highly homologous), S229 is found to be positioned at the interface between the POU-specific domain of one molecule and the POU-homo domain of the other molecule of the homodimer, in direct proximity to the DNA backbone (Figure 2C, [18]). Thus, phosphorylation at this site likely causes steric and electrostatic disruption of this specific Oct4 homodimer-DNA configuration (the PORE homodimer), but not of the MORE homodimer configuration or Oct4 heterodimer complexes.

BLASTing the 15-bp PORE sequence revealed a large cohort of genes potentially regulated by such a mechanism (although osteopontin, the one target gene previously known to be regulated by a PORE sequence, was not identified in this analysis). Previous whole-genome chromatin immunoprecipitation analyses have identified a wide set of Oct4 target genes [6], [28], as well as describing co-occupancy of Oct4 and other transcription factors at many of these genes [6]. Although low, the overlap between our data set and those previously generated (6.2%) was quite similar to the overlap between the previously generated data sets themselves (8.1%, [28]), and may reflect 1) the possibility that only a limited set of Oct4 targets are in proximity to a PORE sequence, and 2) potential low genomic coverage in our search. Additional predicted gene targets were verified to bind Oct4 *in situ*, and targets were found to be enriched in several developmental and molecular processes by gene ontology analysis. Thus, control of homodimer formation could be a major mechanism for regulating transcription of a diverse sub-set of Oct4 target genes.

### Signaling pathways which regulate PORE-dependant transcription

Signaling pathways which contribute to regulation of Oct4 stability and transactivation have not been identified previously; indeed, this area remains a conspicuous “black box” in our understanding of the circuitry which controls pluripotency. In Oct4, S229 is predicted to be phosphorylated by protein kinase A (PKA); therefore, we tested the effects of the PKA activator 8-Br-cAMP and found that stimulation resulted in a rapid and transient increase in Oct4 protein levels in P19 cells (although sustained increases were observed in 3T3 cells, e.g. Figure 4E), with no effect on Oct4 mRNA levels. This increase was accompanied by enhanced chromatin occupancy and PORE-dependent transcription of Oct4 targets (Figure 3D–E).

As a test of this model, that these potential Oct4 phosphorylation events can shape the ES cell transcriptional landscape, Oct4 point mutants in an inducible expression vector can be stably transfected into ES lines which conditionally express wild-type Oct4 [14]. Upon shutting off wild-type Oct4 expression, the effects of these Oct4 point mutations on ES cell proliferation, differentiation potential, and gene expression could be determined. Such experiments would potentially reveal a role for the proposed phosphorylation event in regulating Oct4 activity and provide further insight into how the Oct4 transcriptional network regulates pluripotency.

Several studies have examined Oct4 genetic [6], [28] and protein [33] networks. One important outcome of these studies is the observation that Oct4 and its protein binding partners form complex auto-regulatory circuits in which Oct4, Sox2, and Nanog proteins bind to each other's promoters. This auto-feedback system has been proposed as a feature of robustness, i.e. minor perturbations to the system will not produce major effects on gene transcription. Our data support and extend this idea. Despite highly elevated levels of Oct4 protein following treatment with 8-Br-cAMP, no change in transcription of genes controlled by Oct4 heterodimers (i.e. Oct4 itself, FGF4) was observed. This in turn suggests that stoichiometric control of transcription factor levels may provide robustness to this system. Thus, in the case of genes which are not subject to this stoichiometric, multivariate regulation (i.e. FGF12, osteopontin), absolute increases in levels of the single required protein should be sufficient to enhance transcription. Indeed, we found that putative PORE-containing genes were robustly activated following 8-Br-cAMP treatment (albeit with distinct patterns).

We further demonstrate that the cAMP-responsive enhancement of Oct4 activity is at least partially regulated through the p38 MAP kinase pathway. Previous studies [30], [40] revealed coupling of p38 MAPK signaling to cAMP signaling, which was largely mediated through activation of PKA. We found that specific activation of PKA had moderate effects on Oct4 transactivation; while stimulation of EPAC by itself had little effect on Oct4, simultaneous activation of EPAC and PKA strongly enhanced the effects observed following stimulation of either pathway individually. PKA and p38 MAPK signaling have not generally been explored in regulation of stem cell pluripotency or self-renewal. Our findings that these pathways directly modify Oct4 activity warrant further investigation of these signaling events in control of these processes.

## Materials and Methods

### Cell Culture

P19 cells were cultured in alpha-minimal essential medium (Invitrogen, Carlsbad CA) supplemented with 7.5% calf serum and 2.5% fetal bovine serum (Invitrogen). Differentiation was induced by plating 1×10^6^ cells in a bacterial-grade 10 cm dish with 5% FBS (Invitrogen) and 1% DMSO (Sigma, St. Louis MO). Media was replenished on the second day and aggregates were plated on plastic 10 cm dishes on the fourth day. Media was changed every second day. In some experiments, cytosine arabinoside (Sigma) was added to 5 µg/ml. 3T3 cells were cultured in Dulbecco's modified Eagle's medium (Invitrogen) supplemented with 10% FBS.

### Transfections

For luciferase assays, P19 cells were plated in 24-well plates at 1.0×10^5^ cells/mL in the media described above and transfected with 0.8 µg total DNA containing 400 ng luciferase reporter and 1 ng pRL. In some experiments, 4 ng expression vector was included. Total DNA was held constant by addition of pBSSKII(+). Cells were transfected overnight and media was changed the next day. Indicated drug treatments were started at least four hours after final media change. For 3T3 cell transfection, 1.5×10^5^ cells/mL were plated in 24-well plates in 0.5 mL DMEM+10% FBS. The next day, media was changed to 0.5 mL DMEM (no serum) and cells were transfected with 0.8 µg total DNA, containing 1 ng Oct4 expression plasmid, 100 ng luciferase reporter construct and 25 pg pRL (Promega), and balanced with pBSSKII(+), for four hours followed by addition of 0.6 mL/well DMEM+20% FBS. Media was changed to 0.5 mL/well DMEM+10% FBS the next day. For Oct4-GFP fusion overexpression, cells were plated in 4-well Chamber slides (Nunc) and transfected with 1.6 µg total DNA, containing 0.8 µg Oct4-GFP fusions, as described above. Cells were fixed with 4% paraformaldehyde for 10 minutes and counter-stained with DAPI.

### Luciferase assays

48 hours after transfection, cells were lysed with 100 µL/well 1× passive lysis buffer (PLB, Promega) for 15 minutes with shaking. 5 µL of each lysate was transferred to a white 384 well plate (Corning) and assayed by addition of 25 µL Luciferase Assay Reagent (LAR, Promega) and 25 µL Stop&Glo Reagent (Promega). Data was collected on an Analyst HT 384 well plate reader (LJL Biosystems). In some cases, leftover lysate was spun briefly and mixed 3∶1 with LDS western blot loading buffer (Invitrogen)+β mercaptoethanol, heated at 70°C for 10 minutes and stored at −20°C until use.

### RNA Collection and RT-PCR

RNA was collected using an RNeasy kit (Qiagen) and DNA digestion was performed with RQ1 DNase (Promega). One µg RNA was used for cDNA synthesis with random hexamers (Roche) and SuperScript II reverse transcriptase (Invitrogen). cDNA was amplified using Taq DNA Polymerase (Invitrogen) using exon-flanking and intron-spanning primers. The primer sequences used were as follows:

Oct4up 5′-tggagactttgcagcctgag-3′
Oct4down 5′-tgaatgcatgggagagccca-3′
GAPDHup 5′-accacagtccatgccatcac-3′
GAPDHdown 5′-tccaccaccctgttgctgta-3′
fgf4up 5′-GACACGAGGGACAGTCTTCTGGAG-3′
fgf4down 5′-CCGTTCTTACTGAGGGCCATGAA-3′
OPNup 5′-TTGCGCCACAGAATGCTGTGT-3′
OPNdown 5′- CTGTGGCATCAGGATACTGTTCATC-3′
fgf12up 5′-GGCGATACAGGGTTGAGGAATAG-3′
fgf12down 5′-TGGGACCAAGGACGAAAACAG-3′
mIR-124a2-up 5′-ATCAAGATCAGAGACTCTGCTCTC-3′
mIR-124a2-down 5′-TTCAAGTGCAGCCGTAGGCTC-3′

Samples were run for 19–35 cycles (depending on primer set) with annealing at 58°C and 30 second extensions (60 for Oct4) at 72°C. Densitometry was performed using Kodak MI software (Kodak, Rochester NY).

### Western blot analysis

Cells were washed with ice-cold PBS, lysed for 15 minutes on ice with M-PER protein extraction reagent (Pierce), scraped, and spun at 14,000 rpm for 10 minutes at 4°C. In some experiments, protein concentrations were measured by Bradford assay. Lysates were mixed with LDS loading buffer as described above, and ran on 10% SDS-PAGE gels. Following transfer, PDVF membranes were blocked for one hour with 10% nonfat milk in PBS with 0.1% Tween-20 (PBST) and incubated overnight with primary antibody. The following primary antibodies were used: Oct4 (BD Transduction Laboratories, 1∶1000), phospho-p38 MAPK and total p38 MAPK (Cell Signaling), β-Actin clone AC-15 (Sigma Aldrich, 1∶5000) and GAPDH (Ambion, 1∶40,000). The next days, blots were washed three times for five minutes each with PBST, incubated with HRP-conjugated goat anti-mouse or anti-rabbit secondary antibody (Pierce), washed again, and exposed with ECL reagent (Amersham). Blots were stripped with Pierce Restore western blot stripping buffer for 30 minutes at room temperature.

### Chromatin immunoprecipitation

Confluent 10 cm plates were fixed with 1% PFA at room temperature for 10 minutes, lysed in SDS lysis buffer (50 mM Tris, 10 mM EDTA, 1% SDS, Roche Complete protease inhibitors, pH 8.1), scraped and collected into 1.5 mL microcentrifuge tubes, and DNA was sonicated to 200–800 bp fragments with a Branson Sonifier 250 set to 30% power/90% duty, four 10 second pulses. Tubes were kept on ice for >1 min. between pulses. Samples were spun down at 13,000 rpm for 10 minutes at 4°C and diluted 1∶10 with ChIP dilution buffer (167 mM NaCl, 16.7 mM Tris pH 8.0, 1.2 mM EDTA, 1.1% Triton X-100, 0.01% SDS). Lysates were pre-cleared with Protein A-agarose beads blocked with 2.5 mg/mL sonicated salmon sperm DNA (Sigma-Aldrich) and 0.1% bovine serum albumin (BSA, Santa Cruz Biotechnology). Small aliquots were removed for input fractions. Protein-DNA complexes were then immunoprecipitated overnight with polyclonal Oct4 antibody (sc-9081, Santa Cruz Biotechnology), a mix of monoclonal Oct4 antibodies (BD Transduction Labs, Santa Cruz Biotechnology, Chemicon), or normal serum controls (Pierce) on a rotator at 4°C.

The next day, complexes were isolated by incubation with Protein A beads (described above) for one hour at 4°C with rotation. Beads were washed sequentially in lo-salt buffer (150 mM NaCL, 20 mM Tris pH 8.0, 2 mM EDTA, 1% Triton X-100, 0.1% SDS), hi-salt buffer (500 mM NaCl, 20 mM Tris pH 8.0, 2 mM EDTA, 1% Triton X-100, 0.1% SDS), and LiCl wash buffer (10 mM Tris pH 8.0, 1 mM EDTA, 1% sodium deoxycholic acid, 1% NP-40, 0.25 M LiCl), followed by two washes in ice-cold TE, all for five minutes with rotation at 4°C. Chromatin was eluted in elution buffer (1% SDS, 0.1 M NaHCO_3_), 2×10 minutes. Cross-links were reversed by addition of 200 µM NaCl and heating for 4 hours at 65°C. Proteins were digested with Proteinase K (American Bioanalytical, Natick MA) and DNA was purified by phenol-chloroform extraction and ethanol precipitation. DNA was dissolved in H_2_0 and used for analysis. The following primer sequences were used:

Nanogup 5′-GTCTTTAGATCAGAGGATGCCCC-3′
Nanogdown 5′-CTACCCACCCCCTATTCTCCCA-3′
fgf12up 5′-AAGCCATCTCCCCAGACAAGAATA-3′
fgf12down 5′-GCTGATGGAGCACAATGACTATGA-3′
PDE3Aup 5′-ATCAACCAAAGAGGACACAAGGAG-3′
PDE3Adown 5′-CCCAAAAACTAAAAGAGCAGAGCG-3′

Samples were run for 25–35 cycles at 60°C annealing with 30 second extensions at 72°C. For real-time PCR analysis, 1 µL of chromatin was used as template in triplicate reactions using FastStart SYBR Green Mastermix (Roche, Indianapolis IN) on a CFX96 real-time PCR detection system (Bio-Rad, Hercules CA). C(t)s were automatically assigned by the software and confirmed by manual examination of the fluorescence data. The % of input for each sample was calculated for normalization and the ratio of (% input Oct4)/(% input NRS) for each condition was calculated. Melting curve analyses confirmed the specificity of amplified products.

### Drug treatments

Before all treatments, media was changed four hours before initiation. For proteasome inhibitor experiments, differentiated cells were treated with indicated concentrations for four hours. Undifferentiated cells were treated for 1 hour. Lactacystin, MG-132, and SB202190 were from Calbiochem. 8-Br-cAMP was from Sigma-Aldrich. N^6^-Bnz-cAMP and 8-pCPT-2′-O-Me-cAMP were obtained from Axxora LLC (San Diego CA).

### Bioinformatic analyses

Phosphorylation sites were predicted using Scansite 2.0 (http://scansite.mit.edu; [25]) set on high stringency. For BLAST analysis, the canonical PORE sequence (ATTTGAAATGCAAAT) and an alternate sequence known to bind Oct4 (ATTTGAAAGGCAAAT, [18]) were used with the BLASTN program to query the mouse genomic+transcript database with parameters optimized for short, nearly exact matches with word size set to 15. Gene information, including name and distance from PORE occurrence, was manually curated.

For comparison with lists generated from previous studies, gene identifiers were downloaded and pooled from supplemental data lists, and converted to common identifiers using the DAVID gene ID conversion tool (http://david.abcc.ncifcrf.gov), Matchminer utility (http://discover.nci.nih.gov/matchminer/index.jsp; [41]) and WebGestalt Gene Set Analysis Toolkit (http://bioinfo.vanderbilt.edu/webgestalt; [42]). Multiple conversions were performed to enhance coverage as completely as possible. Analysis of overlaps between the merged ChIP and PORE lists, and resulting gene ontologies, were performed with WebGestalt.

### Molecular modeling

Protein database coordinates for the Oct1/PORE structure reported in ([18], PDB accession #1HF0) were visualized using UCSF Chimera package (http://www.rbvi.ucsf.edu/chimera) from the Resource for Biocomputing, Visualization, and Informatics at the University of California, San Francisco (supported by NIH P41 RR-01081 [43]).

### Statistical analysis

Data are expressed as mean±SEM unless otherwise indicated. T-tests were performed using Microsoft Excel to determine statistical significance of treatment sets. For multiple comparisons, ANOVA was performed, followed by post-hoc Tukey tests, using Graphpad InStat to determine statistical significance. Alpha values were 0.05 except when adjusted by the post-hoc tests.

## Supporting Information

Figure S1 8-Br-cAMP upregulates PORE target genes. Gel images in Figure 3E were quantified by densitometry. Gene expression values were normalized to GAPDH and standardized to untreated levels (time 0). The legend indicates time points in hours.
(0.28 MB TIF)

Table S1 List of PORE genes.
(0.07 MB XLS)

Table S2 Verified Oct4 PORE targets
(0.03 MB XLS)
