# Supplementary figures and images for: Post-Translational Regulation of Oct4 Transcriptional Activity
Source: PLoS One. 2009 Feb 16;4(2):e4467. doi: 10.1371/journal.pone.0004467 (PMC2637973; doi:10.1371/journal.pone.0004467)

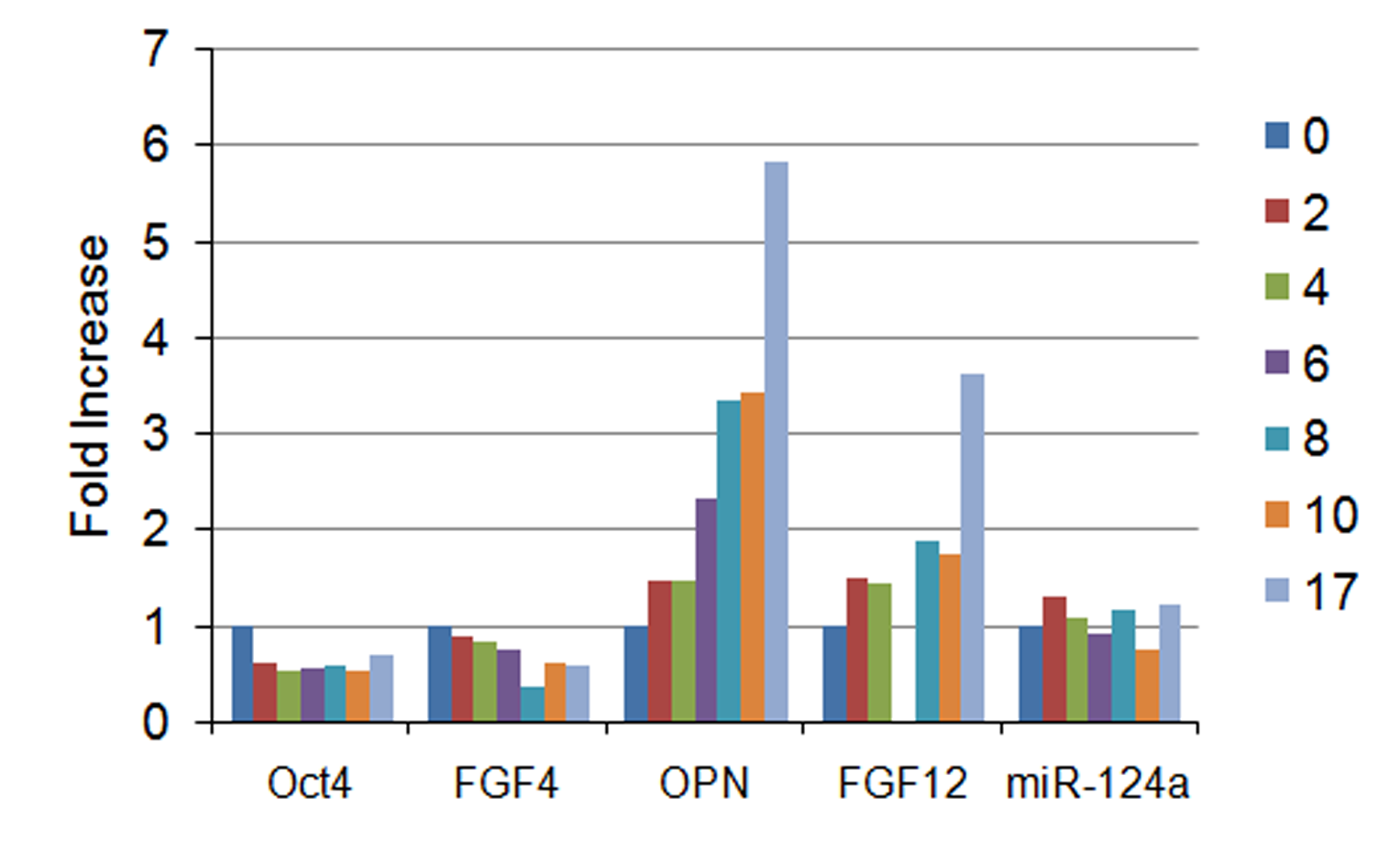

Supplement: Figure S1 — 8-Br-cAMP upregulates PORE target genes. Gel images in Figure 3E were quantified by densitometry. Gene expression values were normalized to GAPDH and standardized to untreated levels (time 0). The legend indicates time points in hours. (0.28 MB TIF) [file pone.0004467.s001.tif]
